# Developmental trajectory of movement-related cortical oscillations during active sleep in a cross-sectional cohort of pre-term and full-term human infants

**DOI:** 10.1038/s41598-018-35850-1

**Published:** 2018-11-30

**Authors:** Kimberley Whitehead, Judith Meek, Lorenzo Fabrizi

**Affiliations:** 1 Department of Neuroscience, Physiology and Pharmacology, University College London, London, WC1E 6BT United Kingdom; 2 Elizabeth Garrett Anderson Obstetric Wing, University College London Hospitals, London, WC1E 6BD United Kingdom

## Abstract

In neonatal animal models, isolated limb movements during active sleep provide input to immature somatomotor cortex necessary for its development and are somatotopically encoded by alpha-beta oscillations as late as the equivalent of human full-term. Limb movements elicit similar neural patterns in very pre-term human infants (average 30 corrected gestational weeks), suggesting an analogous role in humans, but it is unknown until when they subserve this function. In a cohort of 19 neonates (31–42 corrected gestational weeks) we showed that isolated hand movements during active sleep continue to induce these same somatotopically distributed oscillations well into the perinatal period, but that these oscillations decline towards full-term and fully disappear at 41 corrected gestational weeks (equivalent to the end of gestation). We also showed that these highly localised alpha-beta oscillations are associated with an increase in delta oscillations which extends to the frontal area and does not decline with age. These results suggest that isolated limb movements during active sleep could have an important role in experience-dependent somatomotor development up until normal birth in humans.

## Introduction

Isolated limb movements are fundamental for the maturation and mapping of spinal and supraspinal somatomotor circuitry in neonatal animal models^1,2^. The afferent input from these movements is somatotopically encoded in the cortex by alpha-beta neural oscillations nested within a delta wave, anchoring representation in primary somatomotor cortices to the physical layout of the body^3–5^. These alpha-beta oscillations are in fact a hallmark of primary sensory processing in young mammals, across sensory modalities^6,7^. Interfering with these patterns compromises the normal development of cortical maps suggesting a permissive role in cortical development^8^. These brain oscillations are much more likely to be triggered by spontaneous motor activity during active sleep (precursor to rapid eye movement sleep) than during wakefulness^9^, and persist as late as postnatal day 12, which is broadly equivalent to human full-term^9–11^.

Isolated limb movements in very pre-term human infants (30 weeks corrected gestational age (CGA)) evoke similar somatotopically organised alpha-beta oscillations, sometimes over riding slow delta waves, especially during active sleep^12,13^. This suggests an analogous permissive role of these movement-elicited patterns in human cortical development^14^. However it is not known until what developmental stage limb movements fulfil this function.

In this study we investigated (i) whether isolated movements during active sleep are associated with somatotopically organised alpha-beta and delta oscillations in late pre-term and term infants, and ii) at which age they cease to evoke these patterns.

## Results: Movement is Associated with a Localised Alpha-Beta and Delta Power Increase

**Figure 1:**
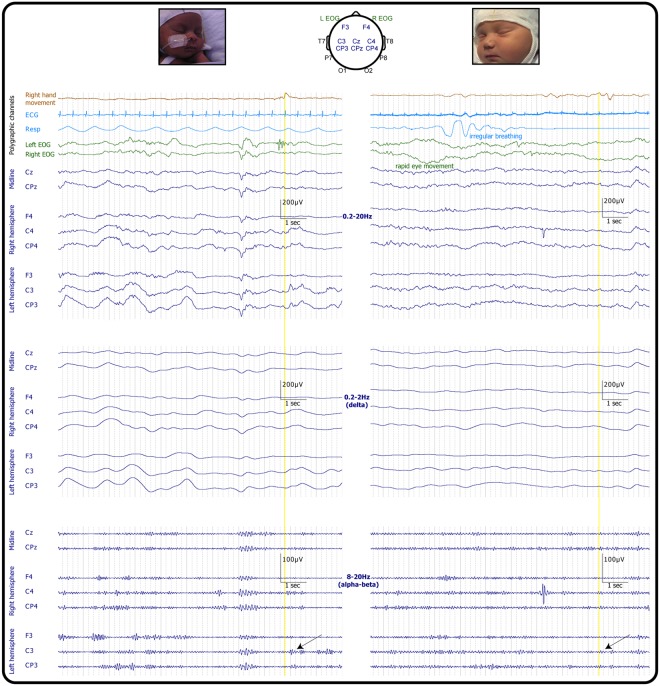
Examples of movement-related localised EEG changes. EEG recording during isolated right hand movement (yellow vertical line) in infants with corrected gestational age 34 weeks (left panels) and 39 weeks (right panels). Both infants are in active sleep, with continuous EEG activity as expected in this sleep state; in these short examples rapid eye movements and irregular respiratory rate and depth are only evident in the older infant. The arrow points to the localised increase in alpha-beta oscillations post-movement over the contralateral central region (C3). Only EEG recordings overlying frontal, central and central-parietal regions are shown for illustrative purposes.

**Figure 2:**
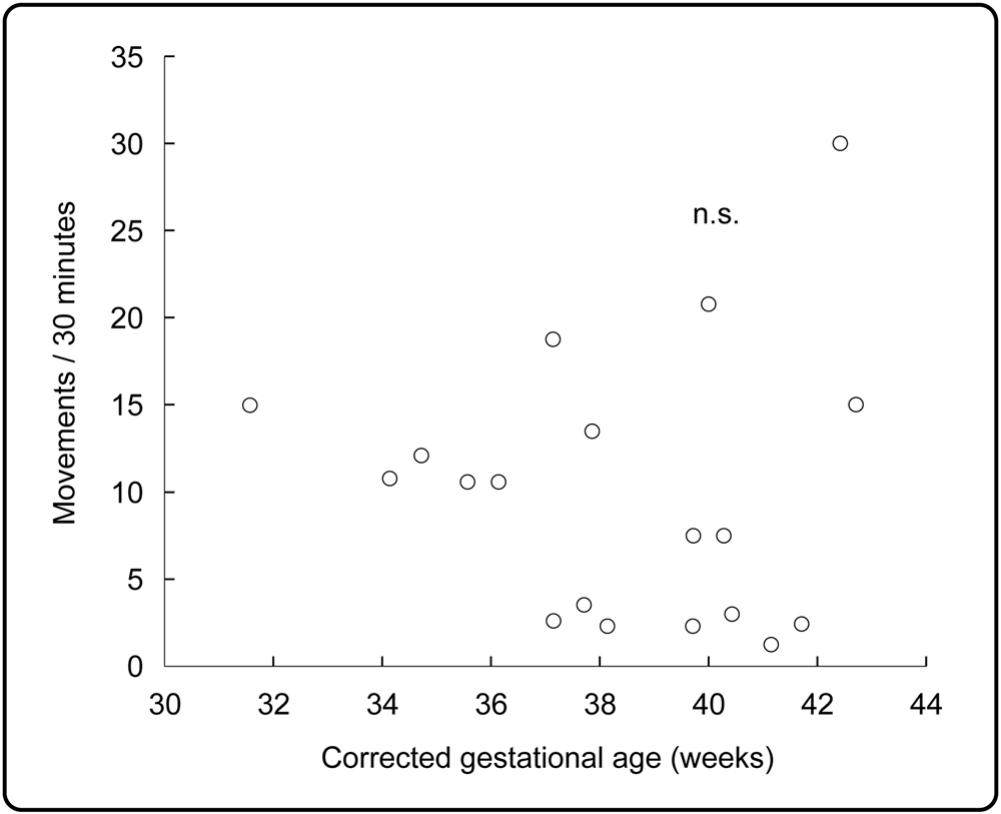
The amount of isolated right hand movements did not change with corrected gestational age. Amount of isolated right hand movements per 30 minutes of active sleep for each of 19 infants.

A total of 143 isolated right hand movements were recorded during active sleep across 19 subjects (Fig. 1; Videos 1 and 4). The number of movements recorded was not associated with CGA (p = 0.518) (Fig. 2).

**Figure 3:**
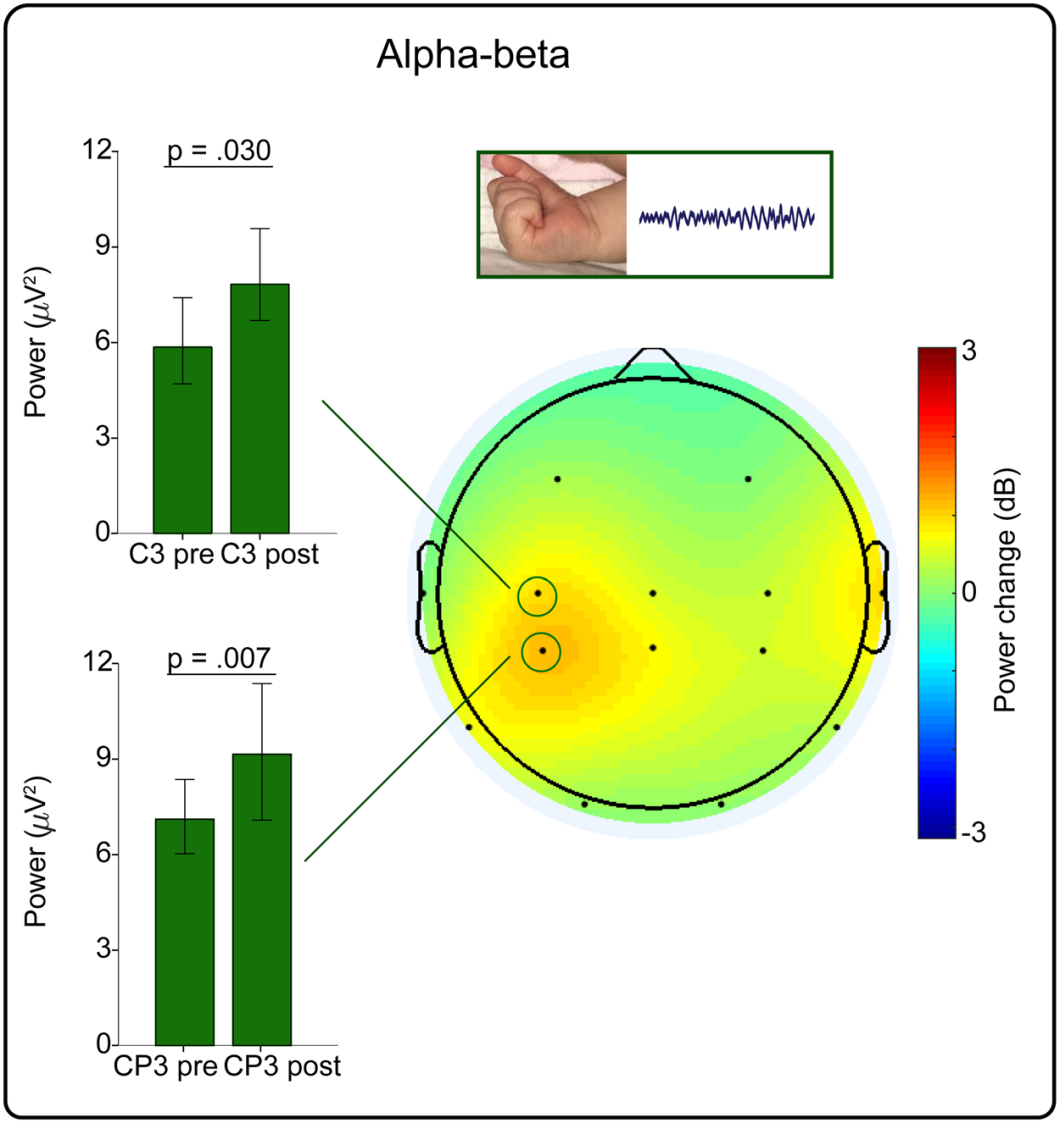
Isolated right hand movements were associated with a localised increase in alpha-beta power. Left: Median (and 95% CI) alpha-beta power pre and post movement at the contralateral central-parietal (CP3) and central electrode (C3). Right: Topographical heat map of the median change in alpha-beta power.

Right hand movements were associated with a significant increase in alpha-beta power localised at the left central-parietal and central electrodes (CP3 p = 0.007 and C3 p = 0.030; 69.2% of movements were associated with a power increase at one or both electrode sites (Figs 1 and 3); all other electrodes p ≥ 0.052).

**Figure 4:**
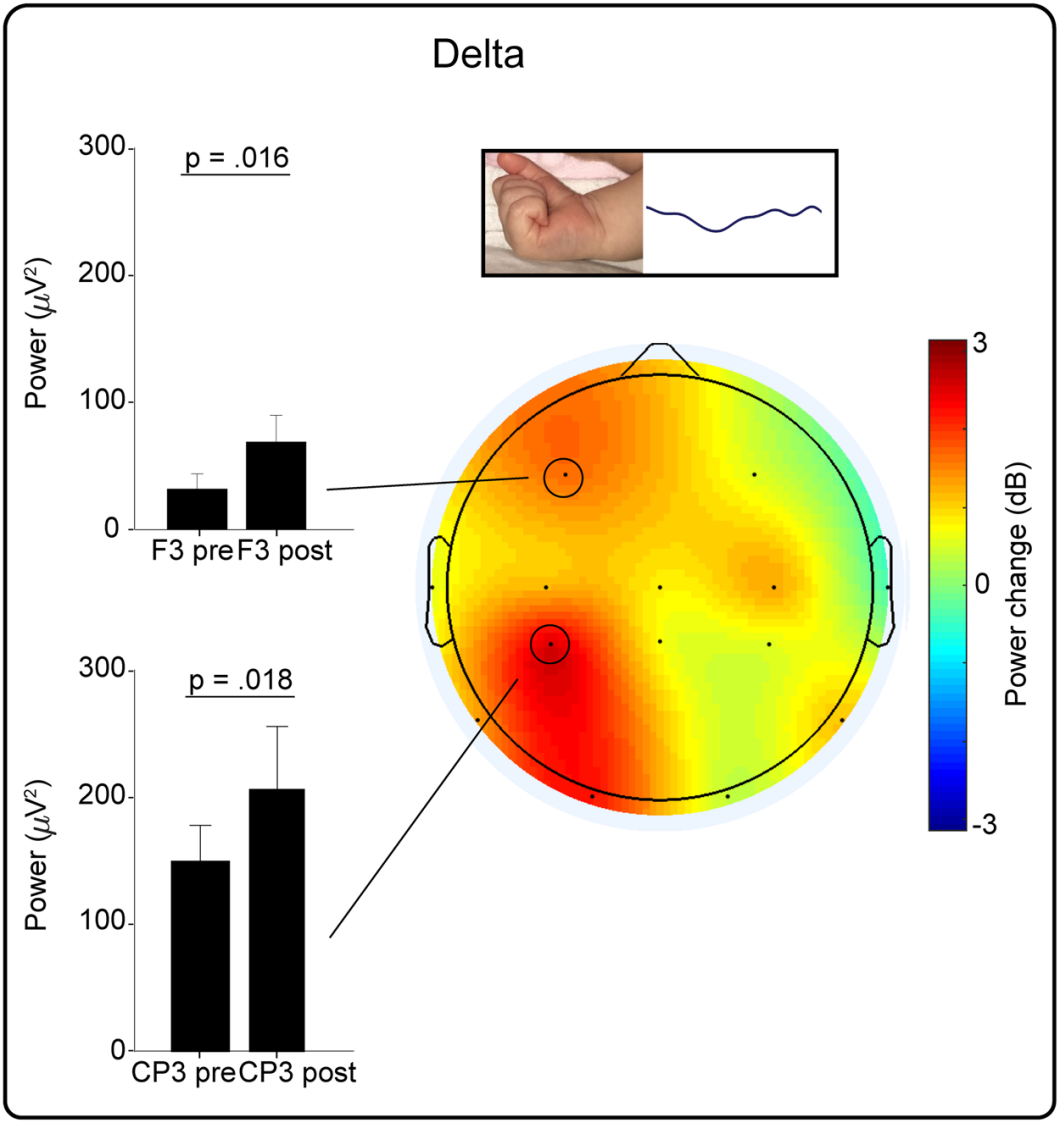
Isolated right hand movements were associated with a localised increase in delta power. Left: Median (and 95% CI) delta power pre and post movement at the contralateral frontal (F3) and central-parietal electrode (CP3). Right: Topographical heat map of the median change in delta power.

Right hand movements were also associated with an increase in delta power over the left frontal and central-parietal area (CP3 p = 0.018 and F3 p = 0.016; 68.5% of movements were associated with a power increase at one or both of these electrode sites (Figs 1 and 4); all other electrodes p ≥ 0.104).

There were no movement-associated changes in the control theta frequency band (p ≥ 0.267 at every electrode).

## Results: Movement-Related Increase in Alpha-Beta Power Declined with Age

**Figure 5:**
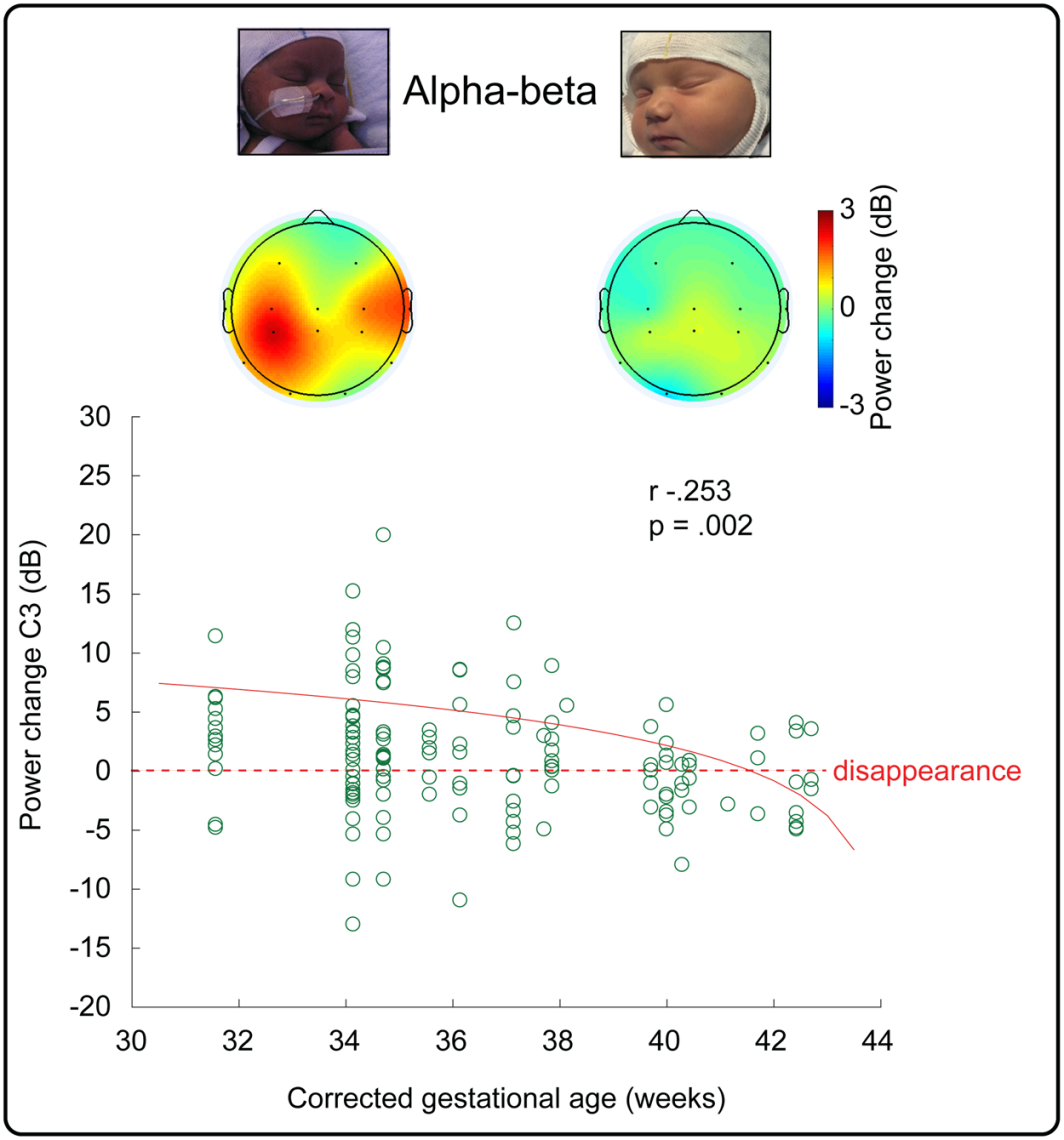
Movement-related increase in contralateral central alpha-beta activity declined with age. Lower panel: Alpha-beta power changes at C3 electrode associated with each movement from 19 infants with best linear fit to the data (solid line, on a logarithmic scale). Top panel: Topographical heat map of the median change in alpha-beta power for younger (left: 31–35 weeks CGA) and older infants (right: 36–42 weeks CGA).

The right hand movement-related increase in left central and central-parietal alpha-beta power declined with CGA and fully disappeared by 41 weeks CGA (C3: r −0.253 p = 0.002, CP3: r- 0.211 p = 0.012) (Fig. 5). The movement-related increase in left frontal and central-parietal delta power did not change with age (CP3 and F3 p ≥ 0.236) (Supplementary Fig. S1).

## Discussion

We have shown that topographically organised movement-related alpha-beta activity is specific to pre- and early-term infants, linearly declines with corrected gestational age, and fully disappears at full-term^15^. This suggests that movement-related alpha-beta oscillations fulfil a role throughout the equivalent of the third trimester of gestation, which is exhausted at full-term age even when the movements themselves persist. These movement-related alpha-beta rhythms are likely to indicate activation of the somatomotor cortex representing the right hand, according to their topographical distribution which overlaps with that evoked by tapping the hand (Supplementary Results). Isolated movements of other limbs are also associated with somatotopically organised increases in alpha-beta activity from 31 weeks CGA (Supplementary Results and Videos 1–3)^12^ and therefore our findings can be likely generalised to other body areas, such as the facial region (Video 5)^16,17^, although the developmental trajectory may differ^18^.

Prior to full-term, alpha-beta oscillations may reflect unique aspects of the immature central nervous system which facilitate sensory cortical development including net excitation within cortical circuits^19^ and amplification of somatosensory input in the spinal cord^20^ or subplate, a transient brain structure that dissolves with development^21,22^. Indeed, the subplate drives alpha-beta oscillations in somatosensory cortex in animal models^8^ and has been associated with alpha-beta activity in pre-term infants^23^. Here we show that movement-related alpha-beta activity overlying somatosensory cortex disappears at full-term age, concordant with reports that other sensory modality-associated bursts of immature alpha-beta oscillations also decline with CGA^24–27^. The timing of the disappearance of movement-related alpha-beta activity at full-term is broadly equivalent to the end of a critical period of experience-dependent somatosensory plasticity in animal models – to which movements contribute^28–30^. Therefore our results suggest that movement-related alpha-beta oscillations occur during an early period of somatosensory plasticity prior to the average time of birth (41 weeks) and may support intra-uterine somatosensory development in preparation for entry into the external world at full-term.

Isolated limb movements occur during the vigilance state that dominates neonatal life - active sleep. Movements during active sleep are unique compared to wakefulness because they affect discrete muscle groups and occur on a background of low muscle tone^9,10^. In addition they are not associated with a corollary discharge – a mechanism which dampens sensory input when it matches a planned movement – so afferent input following these movements is far more likely to evoke cortical activity because it is ‘unexpected’^9,10^. Meanwhile, quiet (non-REM) sleep does not feature any isolated limb movements^16^. Consequently, the high amounts of active sleep present in the newborn period may function to provide optimal input for the developing somatosensory system via frequent isolated movements. Our results show that the function of movement-related alpha-beta oscillations is exhausted at full-term age, in parallel with the onset of a reduction in the amount of active sleep^31–33^.

In contrast to the movement-related increase in alpha-beta activity, the increase in delta activity does not decline with age. This suggests that delta activity continues to subserve a function within somatosensory processing at full-term age^34^, even while overall delta activity declines as resting cortical activity patterns mature^35–37^. We suggest that movement-related alpha-beta activity may diminish first, after refining local primary somatosensory circuits. Meanwhile, movement-related delta activity may persist for longer because it travels across the cortex^38–40^, and therefore facilitates the integration of locally encoded proprioceptive feedback with other associative regions^41–45^. In line with this, we show that movement-related delta activity overlaps with alpha-beta activity in the parietal region, but also extends over frontal areas, which is likely to represent activation of frontal cortex^46^.

This study has some limitations. The variable number of hand movements across infants means that infants with a larger number of movements will contribute more to the results. Further, future longitudinal studies should investigate the influence of extra-uterine experience^35^ and intensive care admission^47^ on movement-related cortical activity patterns. Nevertheless, our results help to build a model of experience-dependent somatosensory maturation in which isolated limb movements contribute to the mapping of somatomotor cortex over the equivalent of the last eleven weeks of gestation, and underline the importance of protecting active sleep in newborns^48–51^.

## Methods

### Participants

Nineteen infants with CGA of 31 + 4–42 + 5 weeks + days were included in this study (Table 1). CGA is defined as gestational age at birth plus postnatal age. Pre-term is defined as <37 weeks^52^. Infants were not eligible for inclusion if they: (i) were receiving sedative, analgesic or anti-seizure medication; (ii) had intra-ventricular haemorrhage (grades I-IV), periventricular leukomalacia, or diagnosed chromosomal abnormality. No infants were on respiratory support or receiving caffeine as a respiratory stimulant at the time of study except for the youngest infant with CGA 31 + 4 weeks + days (high flow oxygen via a nasal cannula; caffeine 5 mg/kg once daily, which does not affect electrical brain activity at this age^53^). Ethical approval was obtained from the NHS Research Ethics Committee, and informed written parental consent was obtained prior to each study. Separate informed written parental consent was obtained to publish photographs of two infants and videos of three infants. The study conformed to the standards set by the Declaration of Helsinki guidelines.

**Table 1:** Demographics of the sample population.

Median (range) CGA (weeks + days)	38 + 1 (31 + 4–42 + 5)
Median (range) GA (weeks + days)	37 + 5 (29 + 4–41 + 5)
Median (range) PNA (days)	2 (1–17)
Sex (% female)	52.6%
Multiple gestation neonates	5.3%

GA indicates gestational age at birth; PNA indicates postnatal age; CGA indicates corrected gestational age: GA at birth plus PNA; Pre-term = < 37 weeks.

### Electroencephalography (EEG) and other physiological recordings

EEG electrodes were positioned bilaterally overlying frontal (F3, F4), temporal (T7, P7, T8, P8) and occipital areas (O1, O2), with high-density coverage overlying central (C3, Cz, C4) and central-parietal areas (CP3, CPz, CP4), according to the modified international 10/10 electrode placement system. The reference electrode was placed at Fz and the ground electrode was placed at FC6/5. Target impedance was <10 kΩ^54^. Electrooculography (EOG) was recorded from electrodes placed laterally to the eyes (F7, F8). Lead I electrocardiography (ECG) was recorded from both shoulders. EEG, EOG and ECG electrodes were disposable Ag/AgCl cup electrodes. Respiratory movement was monitored with a transducer at the thorax (Medifactory). Data were recorded with a direct current (DC)-coupled amplifier from DC-800Hz using the Neuroscan (Scan 4.3) SynAmps2 EEG/EP recording system. Signals were digitized with a sampling rate of 2 kHz and a resolution of 24 bit.

### Experimental protocol

EEG was continuously recorded at rest, and sleep-wake staged as described previously (Table S1 and Fig. S1 in^35^). All recordings included active sleep, which is the dominant vigilance state in pre-term and full-term infants. Active sleep was identified behaviourally according to cot side observation of rapid eye movements, largely irregular breathing, and frequent isolated limb movements. This categorisation was verified offline by assessing the presence of rapid eye movements (EOG), largely irregular respiratory rate and depth (transducer at thorax), and continuous relatively low voltage EEG (Fig. 1)^16,55,56^. EEGs were assessed as normal for age by a clinical neurophysiologist (KW) which included consideration of continuity, defined as uninterrupted electrical activity with <2 seconds of voltage attenuation <25 μV^57^. There was continuous EEG activity during active sleep in all infants, except the youngest with CGA 31 + 4 weeks + days. Isolated movements of the right hand during active sleep were monitored using a movement transducer on the right wrist (n = 17/19 infants) or video (n = 2/19 infants) synchronised with the EEG. When using the movement sensor, isolated movements were identified as deflections exceeding a set threshold and were discarded if there was less than one second of movement-free baseline preceding the movement, or if the sensors at the thorax or shoulders recorded the same displacement (suggesting artefact or widespread movement). All infants were monitored by the experimenter (KW) throughout each study and no generalised ‘startles’ were recorded during active sleep.

### Data pre-processing

Data pre-processing was carried out using EEGLAB v.13 (Swartz Center for Computational Neuroscience) and custom-written Matlab code. Mains interference was removed with a 50 Hz notch filter (4th order Butterworth filter) and, for each epoch, baseline correction was used to remove DC offset. Recordings from electrodes which had poor contact with the scalp were rejected. Missing and discarded recordings were then estimated with spherical interpolation as implemented in EEGLAB.

### EEG power analysis

Data analysis was carried out using EEGLAB v.13, custom-written Matlab code and IBM SPSS version 22. We compared the power content in the alpha-beta (8–20 Hz) and delta (0.2–2 Hz) frequency bands in the 1 second after the movement with that in the 1 second preceding the movement at every electrode (Wilcoxon paired tests). Changes in the theta (4–6 Hz) band were calculated as a negative control. A tapering Hanning window was used in the calculation of the power spectrum to reduce spectral leakage. Data were analysed with non-parametric tests because they were not normally distributed (Shapiro-Wilk test <0.05). Movement-related power changes were expressed in decibels (Eq. 1). Their developmental trajectory against CGA was then assessed (Spearman’s correlation coefficients). Statistical significance threshold was set to 0.05 for all tests.

1 $${(\frac{{E}_{post}}{{E}_{pre}})}_{dB}=10\cdot {lo}{{g}}_{10}\frac{{E}_{post}}{{E}_{pre}}$$

## Electronic supplementary material

Supplementary Information

Video 1: Example of a right hand movement during active sleep in an infant with corrected gestational age 31 weeks.

Video 2: Example of a left hand movement during active sleep in an infant with corrected gestational age 31 weeks.

Video 3: Example of a left leg movement during active sleep in an infant with corrected gestational age 31 weeks.

Video 4: Example of a right hand movement during active sleep in an infant with corrected gestational age 37 weeks.

Video 5: Example of a facial movement in an infant with corrected gestational age 30 weeks.

## Data Availability

The datasets generated during and/or analysed during the current study are available from the corresponding author on reasonable request.
