# Supplementary material for: Developmental trajectory of movement-related cortical oscillations during active sleep in a cross-sectional cohort of pre-term and full-term human infants
Source: Sci Rep. 2018 Nov 30;8:17516. doi: 10.1038/s41598-018-35850-1 (PMC6269518; doi:10.1038/s41598-018-35850-1)
Supplement: Supplementary file 1 — Supplementary Information [file 41598_2018_35850_MOESM1_ESM.docx]

**Supplementary Information**

Developmental trajectory of movement-related cortical oscillations during active sleep in a cross-sectional cohort of pre-term and full-term human infants

Kimberley Whitehead, Judith Meek, Lorenzo Fabrizi


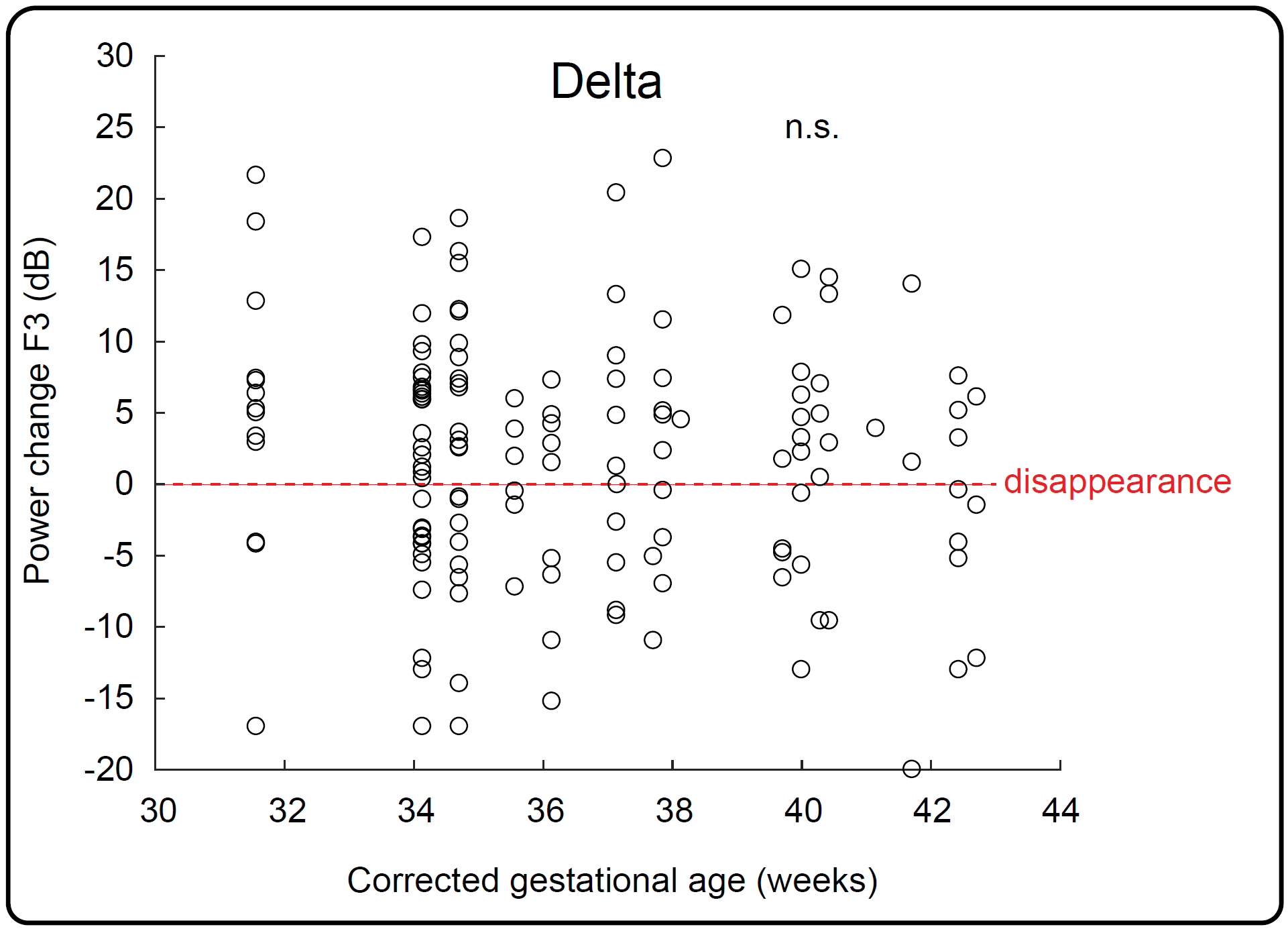


**Supplementary Figure S1: Movement-related increase in contralateral frontal delta activity did not change with age.** Delta power changes at F3 electrode associated with each movement from 19 infants.

**Supplementary Results: Hand and foot movements during active sleep are associated with somatotopically organised increases in alpha-beta power (Videos 1-3)**

Isolated movements of the hand and foot were associated with somatotopically organised increases in alpha-beta power in a single very pre-term infant (31+4 weeks CGA). There were a total of 65 movements: right hand = 13, right leg = 5, left hand = 25, left leg = 22. Right hand movement was associated with an increase in alpha-beta power specifically over the left central region (C3 p = .023; CP3 p = .064; other electrodes p ≥ .101) (Video 1). Right leg movement was associated with an increase in alpha-beta power specifically over the midline central region (Cz p = .043; other electrodes p ≥ .080). Left hand movement was associated with an increase in alpha-beta power specifically over the right central region (C4 trend p = .088; other electrodes p ≥ .135) (Video 2). Left leg movement was associated with an increase in alpha-beta power over the midline central region (Video 3) although not statistically significant (all electrodes p ≥ .108), and an increase in delta power over the midline and temporal-occipital regions (Cz, T7, P7, O1 p ≤ .042). Right leg, right hand and left hand movement was not associated with a change in delta power (all electrodes p ≥ .093). The EEG in all of the videos is displayed with the same sensitivity.

**Supplementary Results: The topographical distribution of the alpha-beta power increase following right hand movements is the same as following taps to the right hand**

After establishing that right hand movement was associated with an increase in alpha-beta power which was topographically organised at C3 and CP3, we demonstrated that taps to the right hand during active sleep, time-locked to the EEG recording, evoked similarly organised changes in a subset of nine/19 infants (n = 225 taps) using identical analysis parameters (alpha-beta increase: C3 and CP3 p <.001; T7 and C4 p <.05; delta increase: trend CP3 p = .064 and C3 p = .078; all other electrodes ≥.167).

**Videos**

Video 1: Example of a right hand movement during active sleep in an infant with corrected gestational age 31 weeks. Note the contralateral increase in alpha-beta and delta oscillations at CP3 and C3. EEG display-filtered: 0.2-20Hz. Scale bar bottom left hand corner. Solid grey vertical lines mark each second and dashed grey vertical lines mark each 200 milliseconds.

Video 2: Example of a left hand movement during active sleep in an infant with corrected gestational age 31 weeks. Note the contralateral increase in alpha-beta oscillations at CP4 and C4. EEG display-filtered: 0.2-20Hz. Scale bar bottom left hand corner. Solid grey vertical lines mark each second and dashed grey vertical lines mark each 200 milliseconds.

Video 3: Example of a left leg movement during active sleep in an infant with corrected gestational age 31 weeks. Note the midline increase in alpha-beta oscillations at CPz and Cz. EEG display-filtered: 0.2-20Hz. Scale bar bottom left hand corner. Solid grey vertical lines mark each second and dashed grey vertical lines mark each 200 milliseconds.

Video 4: Example of a right hand movement during active sleep in an infant with corrected gestational age 37 weeks. Note the contralateral increase in alpha-beta oscillations at CP3. EEG display-filtered: 0.2-20Hz. Scale bar bottom left hand corner. Solid grey vertical lines mark each second and dashed grey vertical lines mark each 200 milliseconds.

Video 5: Example of a facial movement in an infant with corrected gestational age 30 weeks. EEG display-filtered: 0.2-20Hz. Scale bar bottom left hand corner. Solid grey vertical lines mark each second and dashed grey vertical lines mark each 200 milliseconds.
